# Development of Polyphenol-Functionalized Gelatin-Poly(vinylpyrrolidone) IPN for Potential Biomedical Applications

**DOI:** 10.3390/polym14214705

**Published:** 2022-11-03

**Authors:** Lidia Escutia-Guadarrama, David Morales, Daniel Pérez-Calixto, Guillermina Burillo

**Affiliations:** 1 Departamento de Química de Radiaciones y Radioquímica, Instituto de Ciencias Nucleares, Universidad Nacional Autónoma de México, Circuito Exterior, Ciudad Universitaria, Ciudad de Mexico 04510, Mexico; 2 Departamento de Química Analítica, Facultad de Química, Universidad Nacional Autónoma de México, Circuito Exterior, Ciudad Universitaria, Ciudad de Mexico 04510, Mexico; 3 Subdirección de Genómica Poblacional, Instituto Nacional de Medicina Genómica, Ciudad de Mexico 14610, Mexico

**Keywords:** gelatin, poly(vinylpyrrolidone), hydrogel, radiation crosslinking, interpenetrating network, antioxidant

## Abstract

Owing to their suitable physical and chemical properties, hydrogels have been considered a convenient choice for wound dressings because of the advantages that they offer, such as maintaining the moist environment required for wound healing. In this research, interpenetrating hydrogels of polyphenol-functionalized gelatin (GE), a water-soluble protein derived from natural polymer collagen with excellent biocompatibility, no immunogenicity, and hydrophilicity, and polyvinylpyrrolidone (PVP), a hydrophilic, non-toxic, biodegradable, biocompatible polymer that is soluble in many solvents, widely used in biomedical applications, particularly as a basic material for the manufacturing of hydrogel wound dressings, were synthesized. Gallic acid (GA) was selected in this work to study whether the interpenetrating polymer networks (IPNs) synthesized can provide antioxidant properties given that this material is intended to be used as a potential wound dressing. The obtained IPN hydrogels showed improved mechanical properties in comparison with pristine gelatin network (*net*-GE), a porous structure, and good thermal stability for biological applications. The antioxidant capacity of the IPNs functionalized with GA was compared to Trolox standards, obtaining a radical scavenging activity (RSA%) equivalent to a Trolox concentration of 400 µM.

## 1. Introduction

Skin, the largest organ of the human body, serves as the primary barrier for protection against the surrounding environment and external damage, such as exogenous pathogens and mechanical injuries, [1]. For that reason, skin injuries are a health concern worldwide. A wound can be defined as a defect or a break in the skin, or as a result of the presence of an underlying medical or physiological condition [2]. The degree of damage depends on the depth of the wound, which can be superficial, partial-thickness or full-thickness. Wound repair is a physiological process that includes four overlapping phases, namely, hemostasis, inflammation, tissue formation or proliferation, and tissue remodeling [3]. All of these are required to occur in the proper sequence and time in order to achieve successful wound healing. However, there are several factors that interfere with one or more of these phases, resulting in irregular wound healing or even in extra damage [4]. Deficient or inappropriate care of wounds may threaten individual health, triggering infections, other pathological reactions, and even death [5]. 

Due to the risk of complications and infections, different strategies are widely used in the treatment of both acute and chronic wounds; these strategies include, but are not restricted to, the use of wound dressings, which are physical barriers that isolate and protect the wound from the harmful factors of the external environment and further damage [6]. Different kinds of wound dressings are applied on damaged tissue depending on the type, depth, and size of the injury. In general, an ideal wound dressing should meet particular requirements: it can effectively prevent the invasion of bacteria, has a strong liquid absorption capacity but also maintains high humidity at the wound site, and promotes the rapid healing of wounds. Moreover, a wound dressing should possess a certain strength to resist pulling during use, proper adhesion performance, so that it does not cause any extra damage when it is removed from the wound.

Hydrogels are polymeric materials with three-dimensional (3D) crosslinked networks that are capable of absorbing large amounts of water or biological fluids, resulting in materials with unique properties, such as softness, flexibility, high water absorption, and water retention capacity [7]. Owing to their suitable physical properties, hydrogels have become very popular biomaterials for use in drug delivery [8,9], tissue engineering [10], pharmaceuticals [11], and sensors [12,13]. Additionally, hydrogels have been considered a convenient choice for wound dressings because of the advantages that they offer, such as maintaining the moist environment required for wound healing, while at the same time removing the excess wound exudate and promoting wound recovery via granulation and re-epithelialization [14].

Because of their outstanding biodegradability and biocompatibility, natural polymers have been used for decades in the biomedical and pharmaceutical fields. These materials have the capacity to retain relevant biochemical cues, and some of them present structures that can mimic the extracellular matrix of native tissues [15]. Gelatin (GE) is a water-soluble protein derived from natural polymer collagen. It offers many advantages for clinical applications, such as excellent biocompatibility, nonimmunogenicity, and hydrophilicity [16,17]. However, gelatin has poor in vivo mechanical strength and elasticity due to its high swelling capacity in water [18], resulting in the quick breakage of hydrogels. Moreover, gelatin has low thermal stability, which restricts its use in biomedicine [19]. This challenge can be overcome by using crosslinking techniques (physical, chemical, and radiation-induced) and reinforcing materials [18,20].

A number of chemical crosslinking molecules, such as formaldehyde and glutaraldehyde, have been used in attempting to crosslink GE and other polymers. However, it has been documented that the use of these agents results in toxicity issues [17]. Genipin is a naturally occurring crosslinker, and it has been considered an alternative to glutaraldehyde, as it is a nontoxic molecule. Nevertheless, its elevated cost restricts its use [21]. 

Radiation-induced crosslinking is a suitable alternative to crosslinking gelatin molecules without the need for chemical reagents, being a safe and environmentally friendly technique. For that reason, it also ensures that the biocompatibility of the materials is not altered after the crosslinking process [22]. Additionally, it allows the simultaneous sterilization of the irradiated polymer [23,24].

Another method to improve the mechanical strength of hydrogels is to prepare them as an interpenetrating polymer network (IPN); this is defined as a combination of at least two polymer networks in which the polymers have been crosslinked and entangled but not covalently bonded to each other [25]. IPNs obtained from a mixture of synthetic and natural polymers offer the advantage of combining the mechanical properties of the synthetic polymer chain and the biological properties and biocompatibility of the natural component. In the present study, poly (vinylpyrrolidone) (PVP) was chosen as the synthetic polymer to obtain IPN systems. PVP is a hydrophilic, non-toxic, biodegradable, biocompatible linear synthetic polymer that is soluble in many solvents and interacts with many hydrophilic and hydrophobic materials. It has been widely used in biomedical applications, particularly as a basic material for the manufacturing of hydrogel wound dressings [26,27,28].

Gallic acid (GA) is a flavonoid compound also named 3,4,5-trihydroxybenzoic acid. It is found in almost all plants, including fruits, leaves, and wildflowers [29,30], and it has been reported to possess powerful health benefits, such as antioxidant, anti-inflammatory, analgesic, neuroprotective, anticancer, and anti-diabetic properties [31].

Since antioxidants are considered to help control wound oxidative stress and thereby accelerate wound healing, GA was selected in this work to study whether it can provide antioxidant properties to the synthesized IPNs given that this material is intended to be used as a potential wound dressing.

## 2. Materials and Methods

### 2.1. Reagents

Gelatin type A (GE), polyvinylpyrrolidone (PVP), gallic acid (GA), Folin–Ciocalteu phenol reagent (FC), 6-hydroxy-2,5,7,8-tetramethyl-chroman-2-carboxylicacid (Trolox), *N,N,N’,N’*-Tetramethylethylendiamine (TEMED), *N,N’*-Methylenebis(acrylamide) (MBAAm), azobisisobutyronitrile (AIBN), (1-Ethyl-3-(3-dimethylaminopropyl) carbodiimide hydrochloride) (EDC-HCl), *N*-hydroxysuccinimide (NHS), and 2,2-diphenyl-2-picrylhydrazyl (DPPH) were purchased from Sigma Aldrich (Estado de México, México) and used without further purification. Ethanol and methanol were supplied from JT Baker (Ciudad de México, Mexico). Deionized water was obtained from a Milli-Q plus apparatus (Millipore, Molsheim, France). *N*-vinyl-2-pyrrolidone (NVP) and azobisisobutyronitrile (AIBN) were also obtained from Sigma Aldrich. NVP was vacuum-distilled, and AIBN was recrystallized in methanol prior to use.

### 2.2. Synthesis of Hydrogels

#### 2.2.1. Gamma Radiation Crosslinking of Gelatin Hydrogels

Gelatin hydrogels were prepared from 5% and 10 wt% aqueous gelatin solutions. Gelatin type A was dissolved in deionized water at 40 °C with constant stirring. Then, 6 mL aliquots of the solutions were transferred to glass ampoules and bubbled with argon for 20 min in order to eliminate oxygen.

Gamma-ray irradiation of the ampoules was performed at ambient temperature using a Gammabeam 651PT (Nordion International Inc., Ottawa, ON, Canada) ^60^Co gamma-ray source with an average dose rate of 6.1 KGy h^−1^ at various total absorbed doses (5, 10, 15, and 30 KGy) at the Institute of Nuclear Sciences of Universidad Nacional Autónoma de México (UNAM).

The crosslinked hydrogels were washed for 48 h with distilled water, changing the solvent 3 or 4 times a day. Once cleaned, the samples were vacuum-dried at 45 °C until reaching a constant weight. The percentage of reticulation was calculated with Equation (1):

Gel (%) = (W_1_/W_0_) × 100 (1)

where W_1_ and W_0_ are the insoluble crosslinked GE after extraction with distilled water and the initial crosslinked GE, respectively.

The initial formation of the gel dose was estimated using the Charlesby–Pinner equation, shown in Equation (2) [32]:

S + S^1/2^ = (G_d_/2G_c_) + (9.65 × 10^5^/M_n_G_c_) × (1/D_g_) (2)

where S is the soluble part present in the product (if one knows the percentage of the original sample and the insoluble sample after crosslinking, the remanent percentage is the soluble part); G_d_ is the radiochemical yield of scission (the number of chain scissions per 100 eV); G_c_ is the radiochemical yield of crosslinking (the number of crosslinkings per 100 eV); M_n_ is the number-average molecular weight at the beginning of the experiment; and D_g_ is the absorbed dose, converted to kGy from Mrad.

#### 2.2.2. Synthesis of IPN GE/PVP Hydrogels

IPNs were prepared by the free radical polymerization of NVP. MBAAm (2%), TEMED (0.55%), AIBN (3%), and NVP monomer (10% and 15%) were thoroughly dissolved in an ethanol:water mixture (70:30, *v*:*v*). GE crosslinked hydrogels were immersed in 5 mL of this solution for 48 h. After that time, the solutions were bubbled with Ar for 10 min at room temperature, and ampoules were sealed by heat. The polymerization reaction was performed for 4 h in a water bath at 70 °C.

The gels were subsequently washed with an ethanol:water mixture (70:30, *v*:*v*) and vacuum-dried at 45 °C until reaching a constant weight.

#### 2.2.3. Coupling of GA

The carbodiimide-mediated coupling reaction was used to covalently modify the IPN GE/PVP hydrogels with the polyphenol GA according to the procedure of Xu et al. [33] with some modifications. In brief, GA (1 mmol) and EDC-HCl (3 mmol) were dissolved in 10 mL of ethanol in an ice bath. Then, 3 mmol of NHS was added under continuous stirring. After the reaction for 1 h at 4 °C, swollen samples of *net*-GE and IPN GE/PVP hydrogels of approximately 0.0500 g were introduced into the reaction mixture. The reaction was further performed at 4 °C for 1 h and 24 h more at room temperature.

Afterward, the samples were washed with distilled water for 48 h with at least 4 changes of solvent each day to remove the free GA, EDC-HCl, and NHS prior to characterization.

### 2.3. Physicochemical Characterization

#### 2.3.1. FT-IR Characterization

The total attenuated reflectance–Fourier transform infrared (ATR-FTIR) spectra of the different samples were recorded with a Perkin-Elmer spectrum 100 spectrometer (Perkin-Elmer Cetus Instruments, Norwalk, CT, USA) with a universal ATR with a diamond tip, performing 16 scans per sample in the range of 4000–650 cm^−1^. The spectra were analyzed using PerkinElmer Spectrum Spectroscopy software (Version 6.2.0.0055).

#### 2.3.2. Thermogravimetric Analysis

The thermal stability and decomposition temperatures of the hydrogels were measured using a TGA Q50 (TA Instruments, New Castle, DE, USA). Each sample was pulverized and weighted (15–20 mg) before being heated (temperature ramp rate 10 °C/min) under a nitrogen atmosphere in the range of 25–800 °C. The degradation temperature was calculated as the midpoint of the 1st derivative peak of the TGA thermograms for GE, PVP, and IPN GE/PVP.

#### 2.3.3. Scanning Electron Microscopy (SEM)

The hydrogels were immersed in deionized water at room temperature until equilibrium was reached. The swollen hydrogel was then frozen in liquid nitrogen for 10 min and fractured carefully prior to being subjected to lyophilization (Labconco, Kansas City, MO, USA). The interior morphology of a specimen of the freeze-dried hydrogels was observed using scanning electron microscopy (JEOL, Tokyo, Japan). Before conducting the SEM observation, the specimen was fixed on aluminum stubs and coated with gold for 60 s.

#### 2.3.4. Equilibrium Swelling Time and Water Content Test

Hydrogel cylinders of 2 cm in diameter and 1 cm in height were previously dried and studied to determine their water adsorption capacities. The study was performed by immersing the samples in distilled water at room temperature. The mass gain was measured gravimetrically by intervals of time from 5 min until 100 h of immersion; in each measurement, the sample was taken out from the distilled water, placed on an absorbent tissue paper to remove the excess water, and weighted. This was repeated until the weight remained unchanged.

Equation (3) was used to calculate the swelling percentage:

Swelling (%) = [(W_s_ − W_d_)/W_d_] × 100 (3)

where W_s_ and W_d_ represent the weights of the swollen and dry samples, respectively. 

The dried hydrogel samples were placed in 5 mL of distilled water until they fully swelled. They were then blotted with tissue paper and weighted. The water content was calculated according to Equation (4):

Water content (%) = (W_t_ − W_0_)/W_t_ × 100 (4)

where W_t_ is the weight of the swollen hydrogel after reaching equilibrium, and W_0_ is the weight of the dried hydrogel.

All measurements were carried out with triplicated samples and averaged.

#### 2.3.5. Mechanical Testing

The hydrogel samples were characterized using an mechanical microindentation assay as described in [34]. In short, microindentation tests were performed using a commercial microindenter setup, the FT-MTA03 Micromechanical Testing and Assembly System (FemtoTools AG, Zúrich, Switzerland). The force versus displacement data were obtained with a FT-S200 tip (spherical tip with a diameter of 50 µm), with a measurement range of ±200 µN and a maximum resolution of 0.0005 µN (at a sampling rate of 10 Hz). Since the measurements were performed while the hydrogel was submerged in MilliQ water, attraction forces between the hydrogel surface and the tip were not observed. Thus, the Hertz model [34] was used to fit the experimental data after a preprocessing step. The data analysis was performed using Wolfram Mathematica 12.1 (Wolfram Research, Oxford, UK), assuming a Poisson’s ratio of 0.45 [35].

#### 2.3.6. Gallic acid Content

The gallic acid content of the hydrogel–polyphenol conjugates was measured according to the Folin–Ciocalteu method [36,37]. Briefly, triplicated samples of IPNs with gallic acid were immersed in 300 µL of 1N Folin–Ciocalteu phenol reagent solution mixed with 2.4 mL of sodium carbonate solution 0.5 M. The volume was adjusted to 3 mL with 300 µL of distilled water. Samples of *net*-GE without gallic acid were used as controls.

The absorbance of the samples was determined at 750 nm using a UV–Vis spectrophotometer (Specord 200 plus, Analytik Jena, Germany). Solutions of gallic acid with concentrations in the range of 5–50 ppm were used as standards for the calibration curve. The gallic acid content of the hydrogels was expressed as mg gallic acid equivalent per gram of sample (GAE/g sample).

#### 2.3.7. Radical Scavenging Activity

The radical scavenging activity of the hydrogels was determined spectrophotometrically by measuring the decrease in the absorbance of the DPPH radical at 520 nm [38]. In brief, DPPH was dissolved in anhydrous ethanol to prepare a stock solution (100 µM). Samples of polyphenol-modified IPNs were then incubated with 2 mL of DPPH solution at room temperature. The radical scavenging activity (%) of the samples was compared to Trolox, a water-soluble derivative of vitamin E, regularly used as a reference standard for antioxidant capacity. All standards and samples were kept in the dark for 30 min. Absorbance values were measured at 517 nm using a UV–Vis spectrophotometer (Specord 200 plus, Analytik Jena, Germany). The free radical scavenging effect was calculated as follows (Equation (5)):

Radical scavenging activity (%) = [1 − (A/A_0_)] × 100 (5)

where A_0_ is the absorbance of the blank (DPPH solution alone), and A is the absorbance of DPPH incubated with the hydrogels. All tests were performed in triplicate and averaged.

## 3. Results and Discussion

**Figure 1:**
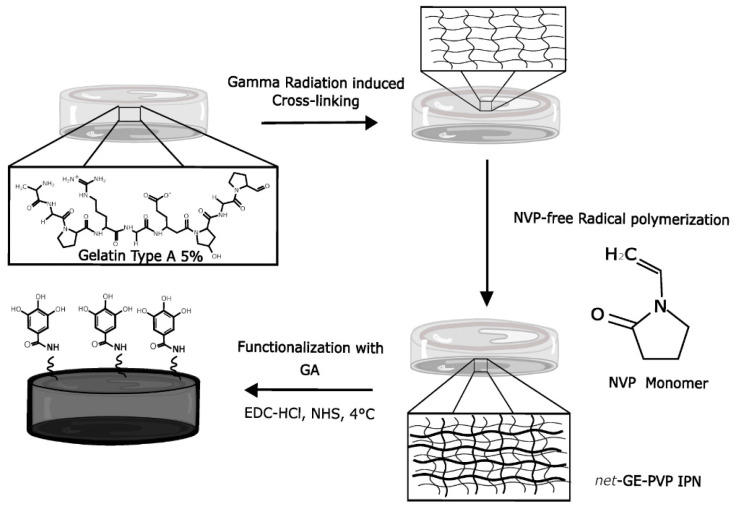
Scheme of the synthesis route to obtain GA-functionalized IPN hydrogels.

Antioxidant IPN hydrogels were prepared according to the global synthesis process depicted in Figure 1. Gamma radiation was used to permanently crosslink GE via the formation of covalent bonds. The resulting *net*-GE hydrogels were interpenetrated with a second network of PVP, synthesized via free radical polymerization with NVP monomers using N,N′-Methylenebisacrylamide (MBAAm) as a crosslinking agent, with N,N,N’,N’-Tetramethyl ethylenediamine (TEMED) as the activator and azobisisobutyronitrile (AIBN) as the initiator. 

### 3.1. Crosslinking of GE

*Net*-GE hydrogels were synthesized via gamma-ray crosslinking in aqueous media. Gamma radiation induces water radiolysis, which yields proton radicals and hydroxyl radicals. According to the proposal of Wan Ishak et al. [22], OH radicals remove H in the gelatin chain and induce the formation of gelatin radicals, which, after recombination, form covalent bonds between gelatin chains.

Four total absorbed doses were selected for this study: 5, 10, 15, and 30 kGy. The hydrogels obtained had differences mainly in their mechanical stability. While the 5 and 10 kGy hydrogels were mechanically unstable and their manipulation was complicated, the hydrogels with a total dose of 30 kGy were brittle. 

### 3.2. Radiochemical Yield Ratios of Crosslinking and Scission

The radiochemical yield ratios of crosslinking and scission (degradation) and the dose of incipient gel D_g_ calculated using the Charlesby–Pinner equation can be observed in Table 1.

The radiochemical yield of the degradation and crosslinking ratio was almost zero, indicating that only crosslinking takes part in this system. Degradation increased with the initial concentration of GE. Due to these results, a 5% GE concentration was chosen to synthesize IPNs and their subsequent characterization.

### 3.3. FT-IR Analysis

**Figure 2:**
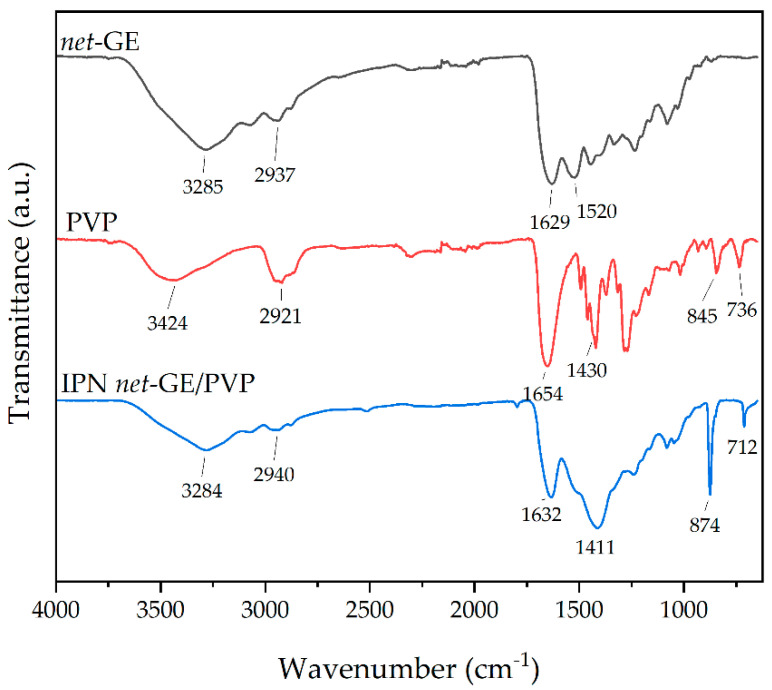
FTIR-ATR stacked spectra.

The FTIR-ATR spectra of GE, PVP, and IPN GE/PVP are presented in Figure 2. GE shows bands at 3285 cm^−1^ corresponding to O-H and N-H stretching, 2937 cm^−1^ corresponding to C-H stretching (methyl groups), 1629 cm^−1^ corresponding to C=O stretching (amide I), and 1520 cm^−1^ corresponding to N-H bending (amide II) [39]. The PVP spectrum shows peaks at 3424 cm^−1^ corresponding to O-H stretching, 2921 cm^−1^ corresponding to C-H stretching (polymer chain), 1654 cm^−1^ corresponding to C=O stretching (pyrrolidone ring), and 1430 cm^−1^ corresponding to C-H bending (polymer chain) [40]. IPN GE/PVP shows the characteristic bands of both GE and PVP, but it is more similar to GE because the quantity of PVP is low, and the two polymers have the same functional groups. Nevertheless, it is possible to identify PVP because this material has a peak at 712 cm^−1^, which is attributed to the C-H rock vibration of polymer long chains.

### 3.4. Thermal Analysis

**Figure 3:**
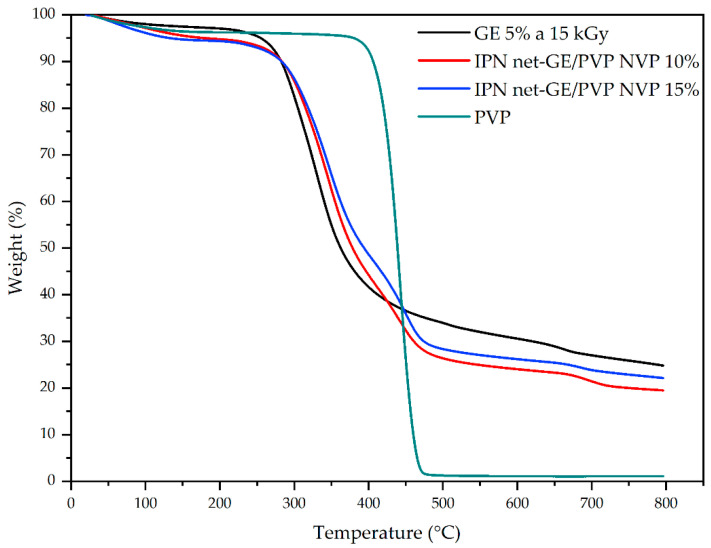
TGA thermograms under N_2_ of *net*-GE, PVP, and IPN *net*-GE/PVP.

In Figure 3, we can see the TGA of *net*-GE, PVP, and IPN *net*-GE/PVP, and it is possible to observe the thermal decomposition steps of each material. GE and PVP show only one step at 330 °C and 442 °C, respectively, while IPN shows two steps at 343 °C and 449 °C, corresponding to starting compounds. This proves that GE and PVP were incorporated into the system in an interpenetrated way; i.e., only physical interactions occurred between both polymers. The thermal stability of IPN is very similar to that of GE. 

### 3.5. Morphology of the Hydrogels

**Figure 4:**
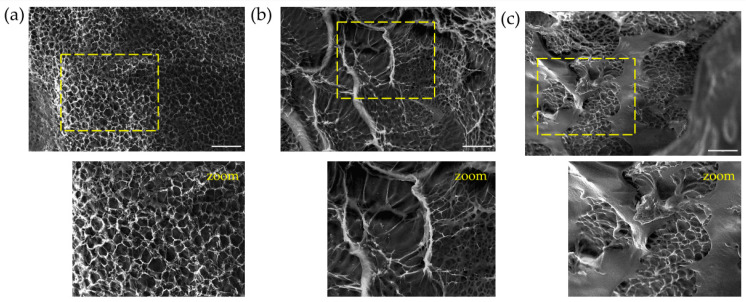
SEM micrographs of cross-section morphology of hydrogels after lyophilization. (**a**) *net*-GE, (**b**) *net*-GE/PVP IPN (10% NVP solution), and (**c**) *net*-GE/PVP IPN (15% NVP solution). Magnification: 1000×. Scale bar: 50 µm.

The SEM micrographs of the freeze-dried hydrogels are shown in Figure 4. Figure 4a corresponds to a sample of *net*-GE crosslinked with a total absorbed dose of 15 kGy. It has a porous structure with a homogeneous appearance. We can observe that a second network is appreciable in the cross-section of the sample IPN *net*-GE/PVP, obtained by the free radical polymerization and crosslinking of NVP 10% and 15% solutions (Figure 4b,c, respectively). It is notable that an increase in monomer concentration generates a reduction in the total void space of the hydrogel structure, which correlates with the decreased values of the equilibrium swelling and an increase in the Young’s modulus value.

### 3.6. Swelling Behavior and Water Content

**Figure 5:**
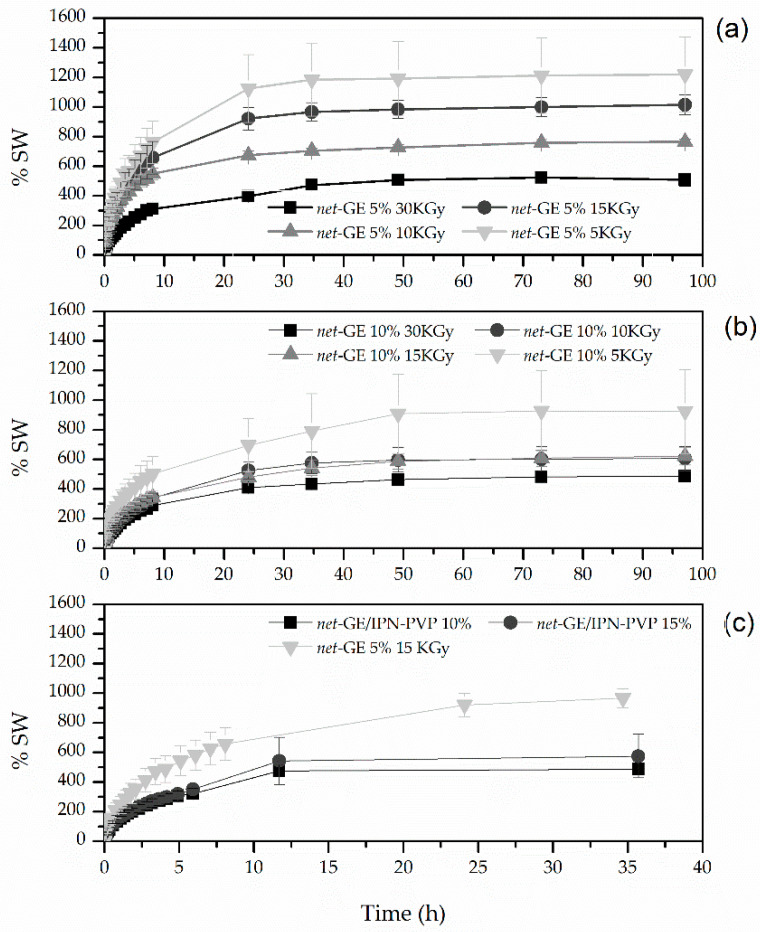
Swelling of *net*-GE synthesized with different GE concentrations: (**a**) 5% and (**b**) 10% in distilled water at room temperature (**c**).

In the equilibrium swelling studies, it was possible to observe different swelling capacities. In Figure 5, we show the effect of radiation dose on the degree of the equilibrium swelling of the GE gels of 5% and 10% concentrations (Figure 5a and Figure 5b, respectively). 

The hydrogels with a total dose of 5 kGy had the highest swelling capacity, around 1220.22 ± 252.43% for GE 5% and 923.86 ± 283.16% for GE 10%; however, their mechanical stability was very poor and decreased with time, resulting in the loss of their integrity after 30 h of being immersed. It is important to clarify that the elevated values of the measurement errors, particularly in the samples of 5 kGy, were due to the loss of material over the course of time. Because of that, the registered values of mass started to differ considerably in the measurements of the samples with more than 20 h of swelling.

The samples of GE 10% irradiated with a total dose of 10 and 15 kGy had very similar behavior. 

In general, the hydrogels obtained from the 10% gelatin solutions had a lower swelling capacity than those obtained from gelatin concentrations of 5%.

The samples with 5 and 10% concentrations of GE and a total dose of 30 kGy were discarded because they had to be manipulated very carefully, as they were very fragile and had the lowest swelling capacity (507.83 ± 42.36% and 485.40 ± 16.00%, respectively).

The water content of all analyzed samples and the equilibrium swelling data are reported in Table 2.

### 3.7. Mechanical Stability Measurements

**Figure 6:**
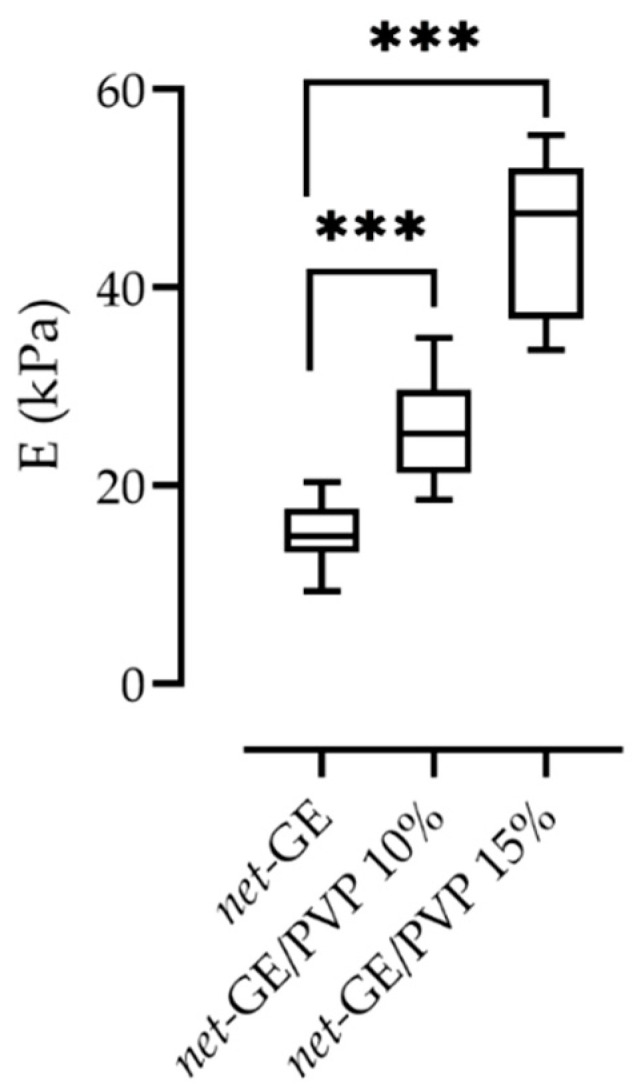
Stiffness of GE-based hydrogels. Young’s modulus was measured using a microindentation test and by fitting the Hertz model to the force–distance curves. A one-way analysis of variance (ANOVA) with Tukey correction was employed for multiple comparisons. It was considered statistically significant when p < 0.05; *** p < 0.0001.

To determine whether the interpenetrated PVP network impacts the mechanical stability of the hydrogels, three different conditions (*net*-GE, *net*-GE/PVP 10%, and *net*-GE/PVP 15%) were mechanically characterized using a microindentation assay (see the Materials and Methods Section). The results of the mechanical characterization presented as mean ± standard deviation of the Young’s modulus (**E**) are reported in Figure 6 and summarized in Table 3. At least two independently manufactured samples per condition were characterized, and at least 10 measurements per sample were performed.

As can be seen in Figure 6, Young’s modulus increases significantly with the increase in PVP content, suggesting a positive correlation between PVP concentration and **E**. These results show that the addition of PVP increases the stiffness of the gelatin network, providing better mechanical stability. The tunability of the mechanical properties of this material, which depend on the total absorbed dose of gamma radiation and the interpenetration of a second polymeric network, is an advantage that allows the gamma radiation crosslinked gelatin-based hydrogels to be suitable for biomedical applications, such as wound dressings and tissue engineering. 

### 3.8. Gallic Acid Content and Radical Scavenging Activity

**Figure 7:**
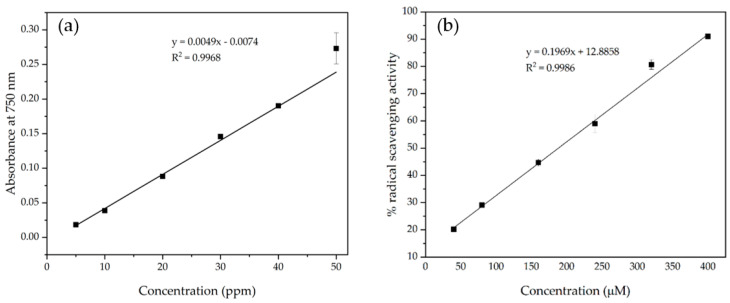
(**a**) Calibration curve of gallic acid; (**b**) calibration curve of Trolox.

The amount of GA attached to the *net*-GE/PVP hydrogels was determined according to the Folin–Ciocalteu method, which is easy to perform, rapid, and low-cost. Polyphenols react with Folin–Ciocalteu reagent in alkaline media to form a blue phosphotungstic–phosphomolybdenum complex that is quantifiable by UV–Vis spectrophotometry at 750 nm. A standard calibration curve of GA, shown in Figure 7a, was used to quantify the amount of GA in the hydrogel samples of known mass employing the Beer–Lambert Law.

Table 3 shows the amounts of GA coupled with the IPN *net*-GE/PVP hydrogels after an amide coupling reaction.

The antioxidant properties of the *net*-GE/PVP IPN hydrogels were investigated using a 2,2-diphenyl-1-picrylhydrazyl (DPPH) radical scavenging assay. Trolox was used as a reference compound to compare the antioxidant efficiency of the prepared hydrogels. 

The DPPH radical has an absorption maximum at 517 nm. Once it is reduced in the presence of an antioxidant molecule, its color changes from purple to colorless due to the formation of stable hydrazine, noted as DPPH-H, upon the absorption of hydrogen from an antioxidant molecule. The antioxidant effect of that molecule is proportional to the decrease in the absorbance values at 517 nm.

The radical scavenging activity (RSA%) is related to the antioxidant activity of the analyzed samples. A calibration curve with increasing concentrations of Trolox, shown in Figure 7b, was used to set a reference of antioxidant capacity and to compare the radical scavenging ability of the hydrogels with that of Trolox standards.

**Figure 8:**
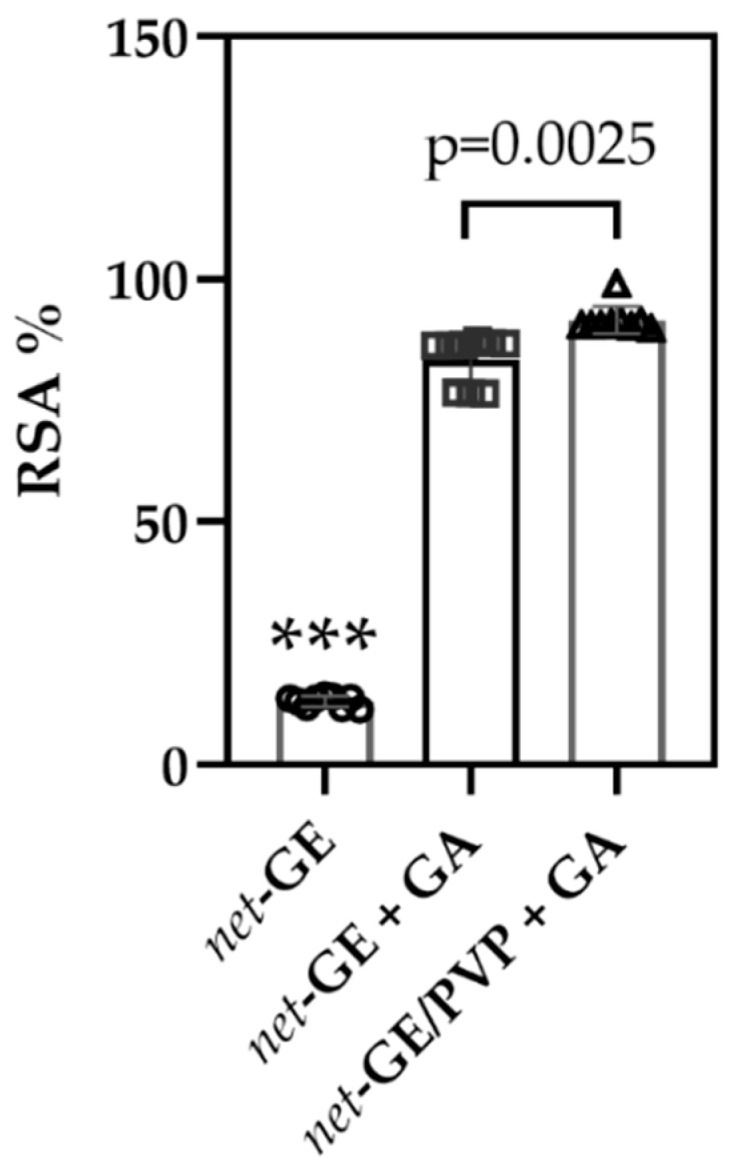
Comparation of radical scavenging activity (RSA%) of *net*-GE/PVP IPN and *net*-GE hydrogels functionalized with GA. *net*-GE was used as a control. A one-way analysis of variance (ANOVA) with Tukey correction was employed for multiple comparisons. It was considered statistically significant when p < 0.05; *** p < 0.0001.

Figure 8 shows that there is a significant difference between the antioxidant capacity of the functionalized hydrogels of *net*-GE and *net*-GE/PVP. Both samples were compared to *net*-GE hydrogels without GA. 

Table 4 shows that strong antioxidant abilities were observed for net-GE/PVP + GA IPN hydrogels, which inhibited 91.61 ± 1.36% of DPPH radical, while *net*-GE + GA showed an inhibition of 83.28 ± 1.22%. Samples of pristine *net*-GE were used as negative controls.

A polymeric material with antioxidant properties is of relevant importance for the development of wound dressings. It has been found that the presence of reactive oxygen species (ROS) in the wound bed promotes a pro-inflammatory environment, which is associated with the oxidative stress of cells and an impaired wound healing process [4]. 

Previous studies have reported that GA exhibits a potent anti-inflammatory effect due to the reduction of inflammatory cell accumulation and the downregulation of major pro-inflammatory cytokines, namely, interleukins, tumor necrosis factor α (TNF-α), and nuclear factor κB (NF-κB) [41,42]. This kind of bioactive material could improve wound management versus the use of dressings, which only protect the wound surface and moisturizes the wound bed, in standard care. In this context, the material studied in this research could potentially restore an appropriate ROS balance, particularly in chronic wounds. 

In the next stage of this research, it is necessary to evaluate the biocompatibility of the material by performing in vitro and in vivo tests. 

## 4. Conclusions

In this study, we developed IPN hydrogels composed of GE and PVP networks, with covalent crosslinking in each network but only physical interactions between them. The crosslinking degree of the *net*-GE hydrogels increased with an increase in the total absorbed dose of gamma radiation, while the equilibrium swelling decreased when the total absorbed dose was augmented. After optimizing the concentration of the initial GE solution and the total absorbed dose of gamma radiation for the GE hydrogels, 15 kGy was selected as the best condition to obtain mechanically stable materials in order to achieve adequate manipulation without breakage. IPN formation was accomplished via the free radical polymerization of NVP inside the crosslinked network of GE, confirmed by thermogravimetric and FT-IR analyses. 

The antioxidant capacity of the functionalized IPNs was evaluated by a DPPH analysis, demonstrating that the synthesized materials had a radical scavenging activity comparable to that of a Trolox standard with a concentration of 400 µM. The results suggest that the polyphenol-functionalized IPN *net*-GE/PVP could be considered as a candidate in the use of polymeric biomaterials for improving the wound healing process.

Further research must be conducted to evaluate the biocompatibility of the materials and their performance in vivo to enhance the wound healing process, and to evaluate their potential as a bioactive material for wound dressing.

## Figures and Tables

**Table 1:** Radiochemical yield ratios and initial gel dose of *net*-GE at different initial GE concentrations.

**Concentration**	5%	10%
**G_d_/G_c_**	−0.0436	0.1115
**D_g_** **(kGy)**	0.2829	0.3978

**Table 2:** Water content and equilibrium swelling of hydrogels of 5% and 10% GE with different total absorbed doses, expressed as percentages.

GE Concentration	Total Absorbed Dose (kGy)	Water Content (%) ^a^	Equilibrium Swelling (%) ^b^
*5%*	*5*	92.22 ± 1.64	1220 ± 252.43
	10	88.43 ± 0.25	764.81 ± 18.28
	15	91.01 ± 0.54	1014.79 ± 68.98
	30	83.49 ± 1.19	507 ± 42.36
10%	*5*	89.76 ± 2.59	923.86 ± 283.16
	10	85.71 ± 1.62	606.12 ± 82.48
	15	86.09 ± 1.22	622.69 ± 61.08
	30	82.01 ± 0.47	485.40 ± 16.00

^a^ Data are shown as means ± SD (*n* = 3); ^b^ data are means ± SD (*n* = 3).

**Table 3:** Elastic modulus of gelatin-based hydrogels.

	E (kPa)
***net*-** **GE**	15.82 ± 2.59
***net*-** **GE/IPN-PVP 10%**	25.71 ± 4.75
***net*** **-GE/IPN-PVP 15%**	44.85 ± 7.55

**Table 4:** Amounts of coupled GA.

Sample	Gallic Acid Content (mg GA/g Sample) ^a^	Antioxidant Activity ^b^
*net*-GE (pristine)	0.20 ± 0.21	13.085 ± 1.22
*net*-GE + GA	1.39 ± 0.19	83.281± 5.85
IPN *net*-GE/PVP	1.64 ± 0.34	91.61 ± 1.36

^a^ Data are shown as means ± SD (*n* = 3); ^b^ data are means ± SD (*n* = 3).

## Data Availability

The data presented in this study are available on request from the corresponding author.
